# Longevity of *Mycobacterium bovis* in Raw and Traditional Souring Milk as a Function of Storage Temperature and Dose

**DOI:** 10.1371/journal.pone.0129926

**Published:** 2015-06-29

**Authors:** Anita L. Michel, Claire Geoghegan, Tiny Hlokwe, Keneilwe Raseleka, Wayne M. Getz, Tanguy Marcotty

**Affiliations:** 1 Department of Veterinary Tropical Diseases, Faculty of Veterinary Science, University of Pretoria, Onderstepoort, South Africa; 2 Mammal Research Institute, Department of Zoology and Entomology, University of Pretoria, Pretoria, South Africa; 3 ARC—Onderstepoort Veterinary Institute, Zoonotic Diseases Section, Onderstepoort, South Africa; 4 Department of Environmental Science, Policy and Management, University of California, Berkeley, CA, United States of America; 5 School of Mathematical Sciences, University of KwaZulu-Natal, Durban, South Africa; 6 Institute of Tropical Medicine, Antwerp, Belgium; Public Health England, UNITED KINGDOM

## Abstract

**Background:**

Unpasteurised fresh and souring dairy products form an essential component of household diets throughout many rural communities in southern Africa. The presence of milk-borne zoonotic pathogens such as *Mycobacterium bovis (M*. *bovis)*, the causative agent of bovine tuberculosis and zoonotic tuberculosis in humans, constitute a public health threat, especially in remote areas with poor disease surveillance in livestock and highly compromised human health due to HIV/AIDS.

**Methods:**

In this study we used culture to determine the longevity of *M*. *bovis* in experimentally inoculated fresh and naturally souring milk obtained from communal cattle in the KwaZulu-Natal province of South Africa. The effect of bacterial load and storage temperature on the survival of *M*. *bovis* was evaluated by spiking mixtures of fresh milk and starter soured milk (aMasi) culture with three concentrations of bacteria (10^2^, 10^4^, 10^7^ colony forming units/ml), followed by incubation under controlled laboratory conditions that mimicked ambient indoor (20°C) and outdoor (33°C) temperatures and periodic sampling and testing over time (0-56 days).

**Results:**

*M*. *bovis* cultured from samples of the fresh and souring milk was identified by PCR analysis. At the highest spiking concentration (10^7^cfu/ml), *M*. *bovis* survived for at least 2 weeks at 20°C; but, at all concentrations in the 33°C treatment, *M*. *bovis* was absent by three days after inoculation. Logistic regression analysis was used to assess the effects of bacterial concentration and time since inoculation, as well as determine the potential half-life of *M*. *bovis* in raw souring milk. Given the most favourable tested conditions for bacterial survival (20°C), approximately 25% of mycobacteria were alive after one day of storage (95% CI: 9-53%), giving an estimated half-life of *M*. *bovis* in raw souring milk of approximately 12 hours (95% CI: 7-27 hours).

**Conclusions:**

This study demonstrates that *M*. *bovis* may survive in fresh and souring milk for periods of time that represent a risk of exposure to people consuming these products, as well as domestic or wild animal populations that have reported opportunities to consume homemade unpasteurised dairy products. The temperature at which the milk is soured and stored substantially affects the survival time of *M*. *bovis*.

## Introduction

In developing countries, inadequate veterinary control measures in combination with poor accessibility to basic veterinary and public health services pave the way for unrestricted and undetected milk-borne transmission of zoonoses [1, 2]. In southern Africa, traditional livestock-owning communities rely heavily for their daily diet on unpasteurised fresh and soured dairy products. Hence, there has been widespread concern that, through the consumption of contaminated milk, neglected zoonoses, such as bovine tuberculosis (BTB) caused by *Mycobacterium bovis*, pose a health risk to both livestock owners and the wider community [3–6].

In studies conducted in Tanzania and in South Africa’s KwaZulu-Natal province, it was established that, respectively, over 90% and 97% (1168/1200) of cattle-owning households reported daily consumption of milk. Fundamental differences, however, were observed in the treatment prior to consumption. While 39% of Tanzanian households reported boiling milk, fewer than 3% of households in the South African study reported heat treatment [7, 8]. Consumption in South Africa was across all age groups (<2 years to >70 years) but primarily by young children and with an overwhelming preference for naturally souring products (aMasi) suggests that dairy consumers are regularly exposed to *M*. *bovis*.

At a continental scale, because the demand for milk is increasing at a higher rate than milk production, the risk of consuming contaminated milk can be expected to become more serious. As a consequence, international efforts are underway to increase the numbers as well as the productivity of livestock [9]. The resulting intensification of dairy farming, however, is a well-known risk factor for BTB [10] with the potential to exacerbate the sporadic or enzootic occurrence of BTB in the majority of countries in Africa [2].

Against this background the reduction of the human exposure risk is an important supplement to test-and-removal strategies in infected herds, because these strategies remain the only effective means to reduce BTB prevalence in cattle [7]. Identifying risk-mitigating controls at the level of consumers requires a better understanding regarding the ability of *M*. *bovis* to survive in conditions that permit regular risk of exposure via household, farming, and food-based activities. This is especially important because traditions and culture greatly influence the consumption of milk and dairy products, 75% of which are produced by the informal sector [9]. In practice, it has been shown that *M*. *bovis* in bovine milk samples is detectable by either culture or molecular methods [11–14]; but an assessment of the health risk posed for consumers necessitates further examination of *M*. *bovis* persistence at infectious levels in local substrates, under a variety of environmental conditions.

Early experiments conducted by Mattick and Hirsch (1946) and Dormer (1953) to determine the longevity of *M*.*-tuberculosis*-complex organisms in souring milk showed that fermenting milk, exposed to elevated outdoor temperatures for parts of the day, produced a detectable but decreasing number of colonies up to five days after inoculation with either *M*. *bovis* or *M*. *tuberculosis*, while cultures and biological assays in guinea pigs remained negative thereafter [15, 16]. In Ethiopia, where the fermented product is stored and consumed for up to 20 days, a recent study found that *M*. *tuberculosis* was present and survived for at least 3 days in milk pooled from skin-test-positive cows. This result suggests that frequent consumption of naturally soured dairy products, derived from infected cows, may be sufficient to expose humans to the infectious dose required for gastrointestinal infection [17].

With the above-described milk consumption practices in mind, our study, involving *M*. *bovis* experimentally-spiked fresh milk, was designed to assess the potential infectivity across time, temperature and bacterial concentrations that mirror likely infection levels and local indoor and outdoor storage conditions in homemade fresh and souring milk (aMasi). Overall we aimed to determine if *M*. *bovis* in dairy products is a potential source of *M*. *bovis* infection in people located in informal farming communities and consuming either unpasteurised fresh milk or souring aMasi products.

## Materials and Methods

### On-farm milk collection and transport

We collected milk from seven cattle-owning households located in three subsistence-based agro-pastoral farming communities in Umkanyakude Health District, KwaZulu-Natal, South Africa, which are situated within 10km of the North-west border of Hluhluwe-iMfolozi Park (HiP). The area is located between 28°00’ and 28°15’ South and between 31°58’ and 32°10’ East. HiP is a provincial wildlife conservation area with documented BTB infections in African buffalo and other wildlife species [18].

Eighteen litres of raw milk were collected, representing a pooled sample from 12 Nguni-crossbred cows. Cows were milked between 4–6 am manually by owners using their routine methods of collection and storage vessels. All seven households milked cattle in outdoor open-air bare-earth kraals, using open plastic containers for milk collection; and for storage they reused plastic or glass bottles formerly containing carbonated drinks. Only one household had access to a communal source of potable water. None of the households had access to permanent electricity, refrigeration or gas cooking facilities. An additional 2.5 litres of unpasteurised souring aMasi were obtained from one of the cattle owning households, to be used as starter culture for the souring process.

A 300 ml aliquot of the milk collected at each household was used for composition, bacterial and antibiotic testing. The remaining milk was pooled into a 20-litre sterile sealable plastic container. All samples were immediately transported in dark, chilled cool boxes to the Tuberculosis Laboratory at the Agricultural Research Council-Onderstepoort Veterinary Institute (ARC-OVI), Pretoria, South Africa, arriving within 10 hours of collection.

Fifteen litres of pooled milk were measured and poured into an open-topped, sterile, plastic drum, and mixed manually using a sterile traditional wooden stirrer with metal wings. Two-and-a-half litres of aMasi were added to the milk to act as a souring starter culture and facilitate spontaneous souring as practiced in rural households. After thorough mixing, 1.65 litres were transferred to each of eight sterile 2-litre glass Schott bottles, labelled to denote the sample number, day, temperature treatment, and bacterial concentration, as described below.

### *M*. *bovis* inoculum and spiking

*M bovis* field strain TB 6630A, used for the experimental inoculation of the fresh milk, was originally isolated from the prescapular lymph node of a naturally infected one-year-old buffalo culled in 2008 at a private game farm in the Mpumalanga province of South Africa. The *M*. *bovis* strain had been genetically characterised as spoligotype SB0130, as well as by a previously reported VNTR (variable number of tandem repeat) typing [19].

A fresh subculture of TB 6630A was prepared in 7H9 medium and, until use, stored at -70°C. Immediately prior to milk inoculation, the culture suspension was defrosted and centrifuged at 15000g for 30 minutes. Cell pellets were re-suspended in saline containing 0.5% Tween 80 at a final concentration of 1 x 10^10^cfu/ml and gently resuspended to ensure that all clumps were dissolved. Ten-fold serial dilutions were prepared from 10^9^cfu/ml to 10^4^cfu/ml.

On arrival at the laboratory the milk/aMasi mixture had been divided into two sets of 4 x 1.65 litre aliquots, with one of the four used as the negative control (no *M*. *bovis* added) and the remaining three respectively spiked at a low (10^2^ cfu/ml), medium (10^4^cfu/ml) and high (10^7^cfu/ml) concentration of *M*. *bovis*. After inoculation, each bottle was sealed with a plastic screw cap and slowly inverted to ensure thorough mixing of the substrate and *M*. *bovis* inoculum. Caps were then loosened to allow the expulsion of gas throughout the souring process. One set of four bottles was then transferred to a temperature controlled room (20°C) and the other set was placed in an incubator (33°C) to mimic ambient indoor and outdoor temperatures, respectively. All samples were processed and placed in the experimental conditions within 4 hours of arrival at the laboratory, and 18 hours after milking.

### Bacterial culture and identification

The survival of *M*. *bovis* over time was determined by the collection of two aliquots of souring milk from each bacterial concentration and temperature condition over 11 sampling times, referred to as ‘time since inoculation’ (TSI). Samples were taken from each concentration and temperature at days 0, 1, 3, 5, 8, and weekly from 2–8 weeks after milking. This timeframe was designed to span and go beyond reported household milk storage duration and anticipated *M*. *bovis* longevity. In addition, each TSI aliquot sample was cultured for 10 weeks to allow time for *M*. *bovis* growth across three consumption periods, including fresh milk (Days 0–2), souring aMasi (Days 3–8) and long-term storage (Day 9 onwards).

At each TSI sampling point, each Schott bottle was inverted to ensure adequate mixing of fat, milk and whey. Then two milk aliquots of 75 ml each were removed and decontaminated by adding an equal volume of 1% cetylpyridinium chloride. Each aliquot was mixed well and incubation at the initial temperature (20°C and 33°C, respectively) was continued for a further week. From each incubation condition, 50 ml of the mixture was then transferred to 3 x 50ml centrifuge tubes and centrifuged for 30 min at 2360 g. The supernatant was carefully discarded to retain milk fat and cream, followed by thorough mixing of the pellet with 25 ml sterile, double distilled water, followed by centrifugation for 10 min at 2360 g. The supernatant was again discarded, leaving approximately 3 ml of cream and pellet. One loopful mixed cream and pellet was inoculated onto each of three Löwenstein Jensen (L-J) media slopes containing sodium pyruvate. The total of 6 slopes per temperature condition and sampling time were incubated at 37°C for a period of 10 weeks and monitored weekly for mycobacterial growth. Isolates were subjected to Ziehl-Neelsen staining and acid fast isolates were subjected to a PCR test targeting genomic region of difference (RD4) for *M*. *bovis* confirmation [20]. As previously described, acid-fast colonies, isolated from the unspiked control vessels that were found to be RD4-negative (no amplification), were confirmed by PCR sequencing of the 16S rRNA as non-tuberculous mycobacteria, [21].

### Milk quality and antibiotic residue analysis

Upon arrival, equal volumes of sub-samples of household milk were pooled (sample 1 = pooled milk from herds 1 to 3; sample 2 = pooled milk from herds 4 and 5; sample 3 = pooled milk from herds 6 and 7) and refrigerated overnight at 4–7°C. They were then submitted for analysis of total bacterial counts, aerobic culture and antibiotic residues by liquid chromatography-tandem mass spectrometry (LC-MS/MS) analysis at the ARC-OVI. All analyses were carried out according to standard operating procedures of the respective accredited laboratories.

### Data analysis

The viability data of the samples were analysed using a logistic regression model, with time of storage and the logarithm of the spiking dose as continuous explanatory variables. Two separate models were used to analyse the microscopy and the PCR data. A culture was considered positive when *M*. *bovis* was detected at least once during the period of observation. Because PCR was not done on all duplicate acid-fast bacilli (AFB)-positive samples, the PCR tested samples were weighted according to the proportion tested. Negative samples included AFB-negative samples, as well as AFB-positive samples that were found to be RD4-negative (no amplification). A non-linear combination of estimators was used to calculate the effect of time on the viability of *M*. *bovis*, using the effect of the spiking dose as a reference. Mean half-life time and 95% confidence intervals were calculated accordingly.

## Results

### *M*. *bovis* survival

A total of 92 samples of stored spiked milk (11 TSI x [3 doses + 1 negative control] x 2 temperatures + 4 inoculation controls) were processed in duplicate (*n* = 184 individual samples) resulting in 552 L-J media slopes that were monitored for *M*. *bovis* growth for a total period of 10 weeks. Confirmation of mycobacterial growth was ascertained by Ziehl-Neelsen staining (ZN) on all six slopes for each TSI and temperature condition, with a subset of ZN positive growth for each confirmed as *M*. *bovis* using RD4 PCR analysis. ZN staining and PCR testing of *Mycobacterium* isolates were performed up to 43 days which was the last TSI at which any colony growth was observed.

If contamination of the entire surface of all media slopes prepared for the same TSI and temperature condition prevented an assessment of whether or not *M*. *bovis* was present in the sample, then the culture result was considered spoiled. Seven out of eight spoiled culture results were observed for milk aliquots sampled at days 8 and 14 from the containers stored at 33°C. All milk sampled from the containers stored at 20°C yielded valid results with the exception of the unspiked samples collected at Day 8 (Table 1).

**Table 1:** Survival of M. bovis over time with regard to spiking dose and storage temperature.

Storage temperature	20°C										33°C									
***M*. *bovis* dose**	10^2^ CFU/ML										10^2^ CFU/ML									
**TSI in days**	0	1	3	5	8	15	22	29	36	43	0	1	3	5	8	15	22	29	36	43
**No. slopes available**	6	6	6	6	6	6	6	6	6	6	NA	6	5	6	0^(S)^	0^(S)^	1	6	6	6
**No. slopes ZN pos**	3	3	1	1	1	0	0	0	0	0	NA	1	0	0	0	0	0	0	0	0
**No. slopes PCR tested**	3	3	1	1	1	0	0	0	0	0	NA	1	0	0	0	0	0	0	0	0
**No. slopes PCR pos**	3	2	1	1	0	0	0	0	0	0	NA	1	0	0	0	0	0	0	0	0
***M*. *bovis* dose**	10^4^ CFU/ML										10^4^ CFU/ML									
**TSI in days**	0	1	3	5	8	15	22	29	36	43	0	1	3	5	8	15	22	29	36	43
**No. slopes available**	5	6	6	6	5	5	6	6	6	6	NA	6	6	6	2	0^(S)^	6	6	6	6
**No. slopes ZN pos**	4	6	6	6	3	0	0	0	0	0	NA	6	0	0	0	0	0	0	0	0
**No. slopes PCR tested**	3	3	3	3	3	0	0	0	0	0	NA	3	0	0	0	0	0	0	0	0
**No. slopes PCR pos**	3	2	2	3	1	0	0	0	0	0	NA	3	0	0	0	0	0	0	0	0
***M*. *bovis* dose**	10^7^ CFU/ML										10^7^ CFU/ML									
**TSI in days**	0	1	3	5	8	15	22	29	36	43	0	1	3	5	8	15	22	29	36	43
**No. slopes available**	6	6	6	6	5	6	6	6	6	6	NA	6	3	3	0^(S)^	0^(S)^	6	6	6	6
**No. slopes ZN pos**	6	6	5	6	1	1	0	0	0	0	NA	6	0	0	0	0	0	0	0	0
**No. slopes PCR tested**	3	4	3	3	1	1	0	0	0	0	NA	3	0	0	0	0	0	0	0	0
**No. slopes PCR pos**	3	3	2	3	1	1	0	0	0	0	NA	3	0	0	0	0	0	0	0	0
***M*. *bovis* dose**	0 CFU/ML										0 CFU/ML									
**TSI in days**	0	1	3	5	8	15	22	29	36	43	0	1	3	5	8	15	22	29	36	43
**No. slopes available**	6	6	6	6	0^(S)^	6	6	6	6	6	NA	6	6	6	0^(S)^	0^(S)^	6	6	6	6
**No. slopes ZN pos**	1	0	1	0	0	0	0	0	0	0	NA	0	0	0	0	0	0	0	0	0
**No. slopes PCR tested**	1*	0	1*	0	0	0	0	0	0	0	NA	0	0	0	0	0	0	0	0	0
**No. slopes PCR pos**	0	0	0	0	0	0	0	0	0	0	NA	0	0	0	0	0	0	0	0	0

Survival of *M*. *bovis* was measured as the number of medium slopes yielding at least one colony of *M*. *bovis* and tested by ZN staining and PCR.

TSI: time since *M*. *bovis* inoculation

*M. *fortuitum* was isolated

^(S)^ Spoiled culture result

*M*. *bovis* was isolated from milk sampled in the cases of all spiking doses and both temperature conditions (20°C and 33°C). Milk stored at 20°C yielded viable *M*. *bovis* up to 2 weeks at the highest concentration of 10^7^ cfu/ml. Lower spiking concentrations stored at the same temperature yielded *M*. *bovis* colonies up to 8 days (10^4^ cfu/ml) and 5 days (10^2^ cfu/ml). At 33°C *M*. *bovis* remained viable at all concentrations for ≥1 but < 3 days. Acid-fast colonies other than *M*. *tuberculosis* complex mycobacteria were detected sporadically during the culture of most milk samples, but the *Mycobacterium* species was not determined. *M*. *bovis* was not isolated from any of the unspiked control samples at any TSI, but *M*. *fortuitum* was isolated and identified, as previously described [23] from the unspiked milk sampled at Day 0 and Day 3.

The presence of acid-fast bacteria was confirmed by Ziehl-Neelsen (ZN) staining for 45 L-J slopes generated by spiked milk stored at 20°C for 1 to 43 days and 13 for spiked milk stored at 33°C. A subset (*n* = 39, 20°C; *n* = 7, 33°C) of ZN positive results was tested by PCR for *Mycobacterium* speciation, of which 31 and 7 were positive, thereby implying that *M*. *bovis* was identified in milk stored at 20°C and 33°C, respectively (Table 1). Eight ZN positive L-J slopes yielded a negative result by PCR. The specificity of ZN was estimated using the data gathered from the spiked milk stored at 20°C and tested between 1 and 43 days. Using PCR as a gold standard and assuming that both methods, ZN staining and PCR, were 100% sensitive, the specificity of ZN staining was found to be 93% (95% CI: 87–97%).

**Fig 1:**
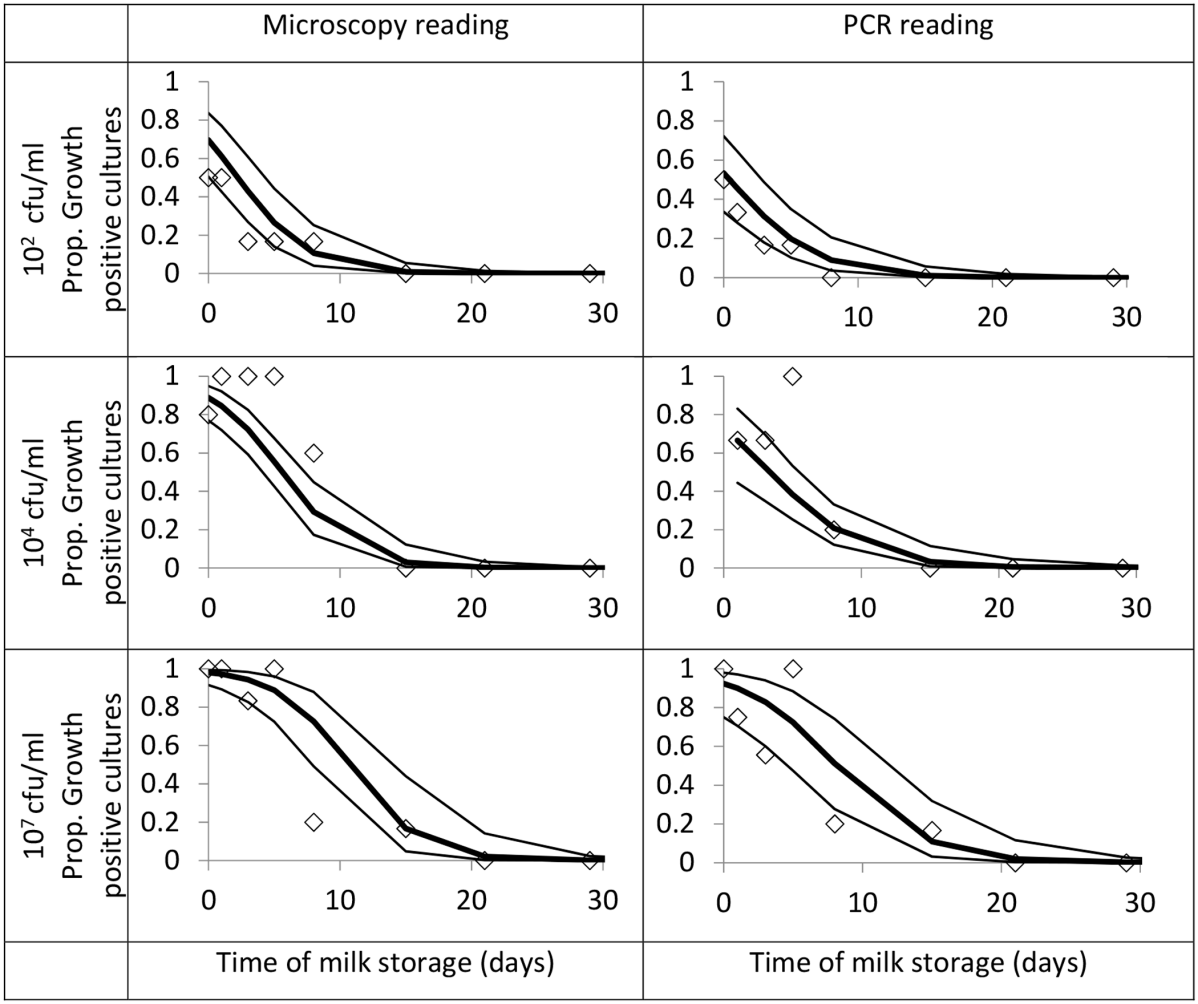
Results of Ziehl-Neelsen staining and PCR amplification of *M*. *bovis* isolated from milk spiked with different doses and stored at 20°C. The left column presents the results using outcome of ZN staining as response variable, whereas a serial combination of microscopy and PCR was used in the right column. Graphs include raw data (◊), estimated means (▬) and the 95% confidence intervals (─). Graphs were truncated on the right because no AFB positive cultures were observed after day 30.

Applying logistic regressions on the data set for 20°C, it was found that the likelihood of isolation decreased with the *M*. *bovis* spiking dose (*p* ≤ 0.002) and also as the TSI increased (*p*<0.001), both for ZN and PCR data. The regression curves, the 95% confidence interval and the raw survival data are presented in Fig 1. The 33°C data was not appropriate for logistic regression because all results were negative for samples incubated for more than 1 day.

The non-linear combination of estimators allowed the effect of the spiking dose to be used as a scale to evaluate the effect of storage time on the viability of *M*. *bovis* in milk at 20°C. One storage day at 20°C had the same effect as an approximately five-fold dilution. The corresponding daily loss of viability, estimated at –75%- 78%, and half-life of 11.1–12.1 hours were calculated assuming a constant loss of viability over time (Table 2).

**Table 2:** Calculation of *M*. *bovis* survival and half-life time (and 95% confidence intervals) using the estimators of two separate logistic regressions applied on ZN and PCR data.

	**ZN**	**PCR**
**Coefficient of TSI (days) [a]**	-0.37 (-0.51, -0.23)	-0.31 (-0.43, -0.18)
**Coefficient of log_10_(dilution) [b]**	-0.62 (-0.94, -0.3)	-0.47 (-0.76, -0.18)
Ratio [c] = [a]/[b]*	0.59 (0.32, 0.86)	0.65 (0.27, 1.03)
**Effect of time using the effect of dilution as a scale [d] = 10^[c]^**	3.93 (2.1, 7.34)	4.46 (1.86, 10.71)
**Viability after 1 day of storage (%) [e] = 1/[d]**	25 (13, 47)	22 (9, 53)
**Daily loss of viability (%) 1-[e]**	75 (87, 53)	78 (91, 47)
Half-life (hours) [f] = 24*ln(0.5)/ln([e])	12.14 (8.34, 22.29)	11.11 (7.01, 26.75)

* Confidence intervals calculated using a non-linear combination of estimators

### Milk quality and antibiotic residues

Total bacteria counts (TBC) in three pooled milk samples ranged from 2.8x10^6^ to >2.5x10^8^ cfu/ml, surpassing national regulated levels of <5 x 10^4^ cfu/ml in raw milk, when no further processing of milk takes place (South African Foodstuffs, Cosmetics and Disinfectants Act, 1972). *Pantoea agglomerans* and *Klebsiella pneumoniae* were isolated from the pooled milk of all herds as the only organisms of potential veterinary significance. *Klebsiella pneumoniae* exhibited resistance to a wide range of antibiotics including, ampicillin, ceftiofur, enrofloxacin, kanamycin, lincospectin, penicillin, polmyxin B, tetracycline and tylosin as determined by the disc diffusion method. One pooled milk sample (sample 1) tested positive (qualitatively) in the microbiological screening test for the presence of B-lactam antibiotics, while analysis by liquid chromatographic quantification for penicillin, cloxacillin, ampicillin, amoxicillin, aminoglycosides, tetracycline and macrolides did not reveal any detectable levels of residues.

Standard compositional analyses were conducted on 3 pooled household milk samples. Lactose levels remained consistent across all pools of herds, but the pooled milk sample obtained from herd 6 and 7 fell below the minimum South African Milk Standards for both fat (2.66%, National standard: 3.25%) and protein levels (3.09%, National standard: 3.43%). The values for the other pooled samples were within accepted norms.

## Discussion

In our study we compared the longevity of *M*. *bovis* in spiked fresh and souring milk at 20°C and 33°C over time and found that both spiking concentration and temperature had an effect on *M*. *bovis* survival. All concentrations yielded viable *M*. *bovis* in milk at both temperatures for ≥1 but <3 days, and during the main aMasi consumption phase, after a souring period of between three and eight days at indoor storage temperature (20°C). The risk decreases for aMasi older than 2 weeks: in this case infectious doses require an initial *M*. *bovis* concentration of ≥ 10^7^cfu/ml in the fresh milk. In addition, fewer people prefer this dairy product compared to younger and less acidic aMasi [22]. However, it should be noted that the common practice of adding fresh milk during the production of aMasi for several successive days offers the possibility of repeated introductions of viable *M*. *bovis* into already ripening aMasi.

A lower storage temperature has been shown to be beneficial for the survival of *M*. *bovis*. It is therefore fair to argue that aMasi produced indoors in traditionally insulated dwellings, or generally during the cooler winter months, constitutes a true source of *M*. *bovis*, provided the aMasi product contains milk from at least one shedding cow. In sharp contrast, aMasi produced at high ambient temperature of 33°C proved free from viable *M*. *bovis* 3 days after the start of the souring process. It was unfortunately not possible for us to statistically quantify the effect of 33°C temperature on *M*. *bovis* survival because of an insufficient number of positive data. The lower longevity of *M*. *bovis* at 33°C could be due to the more rapid pH drop at higher temperatures, as shown by Dormer et al. (1953): they found that the acidity of milk exposed to high ambient temperatures dropped to pH 4.5 on the second day [16]. This can be explained by the faster development of ferments at higher temperature (e.g. 33°C) and hence a steeper pH drop. Both competition with ferments and low pH (< 4 in fermenting milk) affecting the survival of *M*. *bovis* are likely factors that can explain the longer survival of *M*. *bovis* at 20°C compared to 33°C. As a practical conclusion from these findings, we recommend that new batches of aMasi cultures be kept at high ambient outdoor temperature for the first two days and then be kept at ambient indoor temperature beyond two days.

Based on our observation that longevity of *M*. *bovis* was reduced by close to 80% for every storage day at 20°C, we found that our results were in line with those of other investigators. In the study conducted by Mariam (2014) *M*. *tuberculosis* was isolated from milk naturally infected with approximately 10^4^ cfu/ml for ≥3 days but < 7 days of storage at a room temperature varying from 20°C—24°C (Mariam, pers. comm), compared to 8 days at a constant temperature of 20°C in our study involving *M*. *bovis*. This comparison may provide a further indication that sample decontamination prior to culture probably has minimal adverse effects on the viability of *M*. *bovis*, because the mentioned study made use of an antibiotic cocktail in favour of decontamination. Similarly we would expect that alternating the storage temperature between low ambient indoor and high outdoor conditions on a daily basis, as reported by Dormer et al. (1953), would result in an overall similar longevity compared to <3 days at a constant temperature of 33°C. The survival period found by these authors was 5 days; though the dose used for spiking the milk was not quantified, but merely described as “growth of a 16-days-old culture of human tubercle bacilli washed off a Löwenstein-Jensen slant”.

It is estimated that a minimum infective dose of <10 cfu may cause respiratory infection with *M*. *tuberculosis* in humans [23]. Although it must be assumed that a much larger dose in the order of millions of bacilli is needed to achieve oral infection [21 because the anti-bacterial activity of the stomach acid is likely to destroy the majority of tubercle bacilli, it is also true that, compared to other food types, milk is a more effective vehicle for food-borne pathogens. First, the milk fat molecules promote emulsification and protect *M*. *bovis*/*M*. *tuberculosis* cells from destruction. Second, the liquid nature of fresh or soured milk ensures the shortest possible exposure to the acidifying environment of the stomach. Once in the gut the migration of the bacilli through the mucus membrane is facilitated by the general uptake of nutrients [23]. Against this background, a natural dose of >4 orders of magnitude of bacilli were considered sufficient to infect an adult human [17], and even more so infants and young children who are not only more susceptible to tuberculosis infection [24] but who consume milk more regularly than adults [25]. Depending on the level of *M*. *bovis* contamination in milk, which in turn is primarily a function of tuberculous mastitis in cows, the milk of one cow contains sufficient *M*. *bovis* organisms to render more than 100 litres of bulk milk infectious for infants [23]. Hence the inclusion of a hypothetical infectious dose of 10^7^cfu/ml in our experiment may well represent a realistic possibility under circumstances where the lack of BTB control measures in cattle permit the development of advanced and generalised cases of BTB in cows. This is further corroborated by the incidental isolation of *M*. *bovis* from a fresh milk sample submitted to our laboratory and subjected to the same protocol for culture. The milk sample had been collected from a newly diagnosed BTB outbreak of unknown duration (Hlokwe unpublished data).

Moreover, the ability of *M*. *bovis* to survive in fresh and souring milk for at least several days opens the opportunity for transmission to other domestic animal species, such as dogs and in some areas also pigs that are fed on household left-over food including milk [25]. Both these species are free-roaming on community land and have the potential for direct or indirect contact not only with humans but also wild animals. Contaminated milk products may therefore serve as a source of *M*. *bovis* at the wildlife/domestic animal/human interface.

Overall high total bacterial counts were detected in all pooled fresh milk samples, indicating contamination levels exceeding the permissible national level by two to four orders of magnitude. No clinical signs of mastitis were observed in the milked cattle and no typical mastitis pathogens were isolated from the milk samples. Thus we concluded that bacterial contamination originated from outside the udder, including the farmer’s hands, containers used for milking and the animal environment. The latter consisted of a ‘kraal’, which is a small open-air enclosure adjoining household structures, and has partial shade and flooring material comprising soil mixed with cattle dung. In the absence of direct access to running water in all of the visited households, hygienic measures such as washing the soiled udders become impractical and are thus largely lacking. Incidental growth of contaminating bacteria was observed on the LJ slopes inoculated with milk stored at 33°C at a rate of 27.3%, while those medium slopes used for culture of milk stored at 20°C showed a lower contamination rate of 7.3%. This can be explained by the high level of bacterial contamination detected in the pooled raw, milk which resulted in faster multiplication and, hence, higher numbers of surviving contaminants at a storage temperature of 33°C compared to 20°C.

No significant antibiotic residues were detected in any of the pooled milk samples, which was unexpected because many farmers, including subsistence farmers, commonly treat illnesses in cattle with antibiotics available over the counter (e.g. tetracycline) as a first choice before seeking professional advice. This lack of antibiotic residues might be an indication of financial constraints or possible lack of attention to livestock health among these farmers.

## Conclusion

Fresh and souring milk is able to facilitate bacterial longevity and hence to serve as a source of zoonotic tuberculosis to consumers, though *M*. *bovis* particles tend to die out rather than multiplying in milk and souring milk. In the light of the high frequency of dairy consumption in rural households, the findings of this study highlight the urgent need to strengthen BTB control programmes in communal cattle. They also highlight the need for locally-relevant, practical and culturally acceptable interventions that minimise the risk of *M*. *bovis* transmission and milk safety in general. Keeping fresh milk in a warm environment for a few days instead of a cool or a refrigerated place may, paradoxically, reduce the *M*. *bovis* load.
